# Influence of Personal, Academic, Social, and Level of Physical Activity Variables on Emotional Intelligence

**DOI:** 10.3390/children9020286

**Published:** 2022-02-18

**Authors:** Iago Portela-Pino, José Domínguez-Alonso, Myriam Alvariñas-Villaverde, Juan José Chinchilla-Mira

**Affiliations:** 1 Department of Health Sciences, Faculty of Health Sciences, Isabel I University, 09003 Burgos, Spain; iagoportt92@gmail.com; 2 Research Group on Education, Physical Activity and Health (GIES10), Galicia Sur Research Institute (IIS Galicia Sur), SERGAS-UVIGO, 36312 Vigo, Spain; jdalonso@uvigo.es; 3 Department of Psycho-Socio-Educational Analysis and Intervention, Faculty of Educational Sciences and Social Work, University of Vigo, 32004 Ourense, Spain; 4 Department of Special Didactics, Faculty of Education and Sport Sciences, University of Vigo, 36005 Pontevedra, Spain; 5 Department of General Didactics and Specific Didactics, Faculty of Education, University of Alicante, 03690 Alicante, Spain; jj.chinchilla@gcloud.ua.es

**Keywords:** adolescence, emotional health, person, social skills

## Abstract

In the diverse and complex society in which we live, the support that an appropriate emotional intelligence can provide to adolescents to achieve a satisfactory, balanced, and peaceful coexistence is increasing. The aim of this research was to determine whether personal, academic, and social variables influenced emotional intelligence in adolescent populations. A descriptive-inferential study was carried out with 964 students of compulsory secondary education (*M* = 14.18; *SD* = 1.28), applying the emotional intelligence scale Trait Meta-Mood Scale (TMMS-24). The results show better emotional attention in boys who practice physical exercise and have good social skills; better emotional clarity in girls who practice physical exercise and have good social skills; and better emotional repair in girls under 13 years of age, who practice physical exercise, have a good academic record and good social skills. In conclusion, a solid and specific knowledge of the personal, academic, and social variables that may influence the development of emotional intelligence in the adolescent period allows helping students in the prevention or modification of undesirable aspects they may have in their relationships with society.

## 1. Introduction

In recent times, there has been a concern to study issues related to students’ emotional well-being [1]. The continuous social, economic, and cultural changes have also had an important impact on educational centers and their students. In order to face these constant mutations, emotional intelligence (EI) is one of the most relevant aspects to favor and facilitate the achievement of goals in the individual’s life project [1,2].

Currently, no one doubts that EI significantly influences the personal, academic, and social aspects of individuals, especially in the adolescent period [3,4,5,6,7]. Good emotional management and adaptation will allow positive relationships, while its absence can lead to problems in the social and school environment in the individual. In fact, Soriano and Osorio [8] and Bisquerra et al. [9] argue that it would be desirable for adolescents to acquire a comprehensive development through emotional skills to build a strengthened personality and self-esteem and that, consequently, the students themselves learn to manage their feelings, resolve conflicts, make decisions, and opt for attitudes that promote health and well-being in all senses and areas of life.

In the educational field, there are many ways or models to consider EI, but the one with the greatest theoretical and scientific support is the ability model of Mayer and Salovey [10]. He conceptualizes EI as “an ability to perceive, appraise, and express emotions accurately; the ability to access and/or generate feelings that facilitate thinking; to understand emotions and reason emotionally; and finally, the ability to regulate one’s own and others’ emotions” [10] (p. 10). Based on this operational definition, EI would be symbolized by three dimensions: the perception of emotional states, an appropriate understanding of the affective nature, and the capacity for emotional regulation as a way of personal growth. Thus, EI becomes a capacity for abstract reasoning, from a set of emotional symbols, in order to find a correct solution to a problem, varying from one individual to another [11,12].

If we pay attention to personal determinants, gender is known to be a differentiating factor. Women obtain higher scores in emotional attention, and men achieve higher scores in emotional repair [13,14]. Similarly, Salavera and Usán [15] observe better emotion management in boys in terms of attention and regulation but a lower understanding of those emotions. However, research such as that conducted by Ruiz and Carranza [16] found no gender differences with respect to self-knowledge or self-control of emotions. Additionally, recent systematic reviews have related physical-sports practice or exercise to EI or its different factors [17,18]. There are also numerous studies that relate EI to sports performance, sports practice, or health. In some cases, it is about studying the relationship between EI and participation in sports and the types of sport practiced [19], where there is a clear relationship between emotions, physiological and psychological stress. EI has also been studied as one of the factors related to aggressive behaviors in sport [20]. Martínez-González et al. [21] establish that high levels of EI are significantly related to good mental health, while low levels are related to certain psychological disorders.

There are also some interesting relationships to note in this regard at the academic level. EI has aroused great interest as a way to improve the cognitive development of students. Recently, the literature has shown that deficiencies in EI skills affect students inside and outside the school context [22]. Consequently, the problems in the educational context associated with low levels of EI have repercussions on students, especially in terms of a deficit in their levels of well-being and psychological adjustment, a decrease in the quantity and quality of interpersonal relationships, a decrease in their academic performance, the appearance of disruptive behaviors, and the consumption of addictive substances [2,23,24,25]. In their study, Rivers et al. [26] argue that the relationship between EI scores and school problems was moderately high, suggesting that adolescents with higher EI have better attentional levels and fewer learning problems.

Nowadays, there are numerous emotional education programs that show an improvement in students’ academic performance and that reinforce the link between EI and academic success [9,27,28,29,30]. In fact, Perera and DiGiacomo [31] have found that the relationship between trait EI and academic performance decreases as the educational level increases, i.e., it is higher in primary than in secondary school and higher in secondary school than in university. Numerous studies support a strong association between academic success and the main dimensions of EI [32,33].

On the other hand, extracurricular activities have been linked to certain emotional skills such as better adaptive behavior and emotional regulation or positive emotional states, as proved by Metsäpelto and Pulkkinen [34] and Hansen et al. [35].

EI also becomes a determinant for personal and interpersonal behaviors [36]. Emotionally intelligent people are not only more adept at perceiving, understanding, and managing their own emotions but are also better able to extrapolate their perception, understanding, and management skills to emotions in others [37]. A new framework for investigating social and emotional adaptation is provided since EI plays an elemental role in the establishment, maintenance, and quality of interpersonal relationships. Consequently, a student with high EI can be a more skilled person in perceiving and understanding other people’s emotions and possess better regulation skills. In fact, a significant relationship is established between social skills and EI [38,39].

In short, EI is a notion with an interventionist tendency in socio-educational environments, which can help to deploy various skills suitable for the personal, academic, and social development of individuals. Consequently, the aim of this research was to determine whether personal (gender, age, physical exercise), academic (school year, academic record, extracurricular activities), and social (social skills) variables influenced EI in adolescent populations.

Therefore, taking into account the adolescent period, the following research hypotheses emerge:

**Hypothesis** **1** **(H1).** *“It is predicted that personal variables (gender, age, and physical exercise) present significant differences in emotional intelligence (attention, clarity and emotional repair).”*

**Hypothesis** **2** **(H2).** *“It is inferred that emotional intelligence (attention, clarity, and repair) is significantly influenced by school variables (school year, academic record, and extracurricular activities).”*

**Hypothesis** **3** **(H3).** *“It is predicted that social skills present significant differences in emotional intelligence (attention, clarity, and repair).”*

## 2. Materials and Methods

### 2.1. Participants

A random sample of 964 compulsory secondary education (CSE) students participated in this study. Their chronological age ranged from 11 to 16 years (*M* = 14.18; *SD* = 1.28). Of the sample, 51.7% were female and 48.3% male. In terms of age groups, 35.2% were under 13 years old, 44.6% between 13 and 15 years old, and 20.2% over 15 years old. In terms of physical exercise, 23.9% hardly do any, 48% do moderate exercise, and 28.1% do it regularly. In the school year, 31.4% attend 1st CSE, 20.5% 2nd CSE, 35.6% 3rd CSE, and 12.5% 4th CSE. In terms of academic record, 30.8% were low, 48% medium, and 21.2% high. In terms of extracurricular activities, 82.2% attended, and 17.8% did not attend. In terms of social skills, 25.9% were low, 49.2% moderate, and 24.9% high.

### 2.2. Instrument

The Trait Meta-Mood Scale (TMMS-24) [40,41] was applied, which measures EI perceived on a point-by-point basis: attention, clarity, and repair [42]. It assesses individual differences according to the degree of individuals’ awareness of their own emotions, as well as their ability to regulate them. It has three dimensions: attention to feelings, which measures the degree to which people believe they pay attention to their emotions and feelings (e.g., “I constantly think about my mood”); emotional clarity, which refers to how people believe they perceive their emotions (e.g., “I am often wrong about my feelings”; and emotion repair, understood as the ability to interrupt and regulate negative emotional states and prolong positive ones (e.g., “Although I sometimes feel sad, I tend to have an optimistic outlook”). It is a Likert scale consisting of 24 statements with five response alternatives, in each of which the respondent has to indicate the degree to which he/she considers his/her emotions and feelings to be those listed in the statement. The students who complete the questionnaire must respond by indicating their degree of agreement with the expression contained in each of the items on a scale ranging from 1 (I do not agree at all) to 5 (I totally agree). Salguero et al. [43] also tested and validated the Spanish version of the TMMS for adolescents.

Likewise, personal and academic variables are collected in an ad hoc questionnaire that gathers gender, age, school year (1st CSE, 2nd CSE, 3rd CSE, and 4th CSE), academic record, and extracurricular activities attendance (yes and no).

Some variables have subsequently been recoded to obtain certain results: for example, age groups (<13 years, 13–15 years, >15 years) or physical exercise groups (low, moderate, and high).

Physical exercise practice is achieved through the Physical Activity Questionnaire for Adolescents (PAQ-A), which recorded the activity carried out in the last 7 days during leisure time, physical education classes, after-school hours, and weekends. In addition, it recorded whether any illness or other event hindered the physical activity or sport practice. The overall result of the test is a score from 1 to 5 points that allows graduation in the level of physical activity performed [44]. PAQ-A was validated for the Spanish population [45,46].

The Social Emotional Competence Questionnaire (SECQ) by Zhou and Ee [47] was used to assess social–emotional skills, establishing cut-off points (low, moderate, high) with the 25% and 75% percentiles. The instrument consisted of 5 factors organized into 25 items on a Likert-type scale from 1 to 6; 1 meant strongly disagree with the proposed statement, and 6 meant strongly agree. The factors were self-awareness (items 1–5), social awareness (items 6–10), self-management (items 11–15), relationship management (items 16–20), and responsible decision making (items 21–25). Social skills have subsequently been recoded into three groups for analysis: low, moderate, and high.

### 2.3. Procedure

All individuals participated voluntarily and anonymously in the study, and all ethical procedures for data collection were respected. The administration of the instrument to the students was done by means of a Google Drive form sent by e-mail, noting the anonymous, voluntary participation, and the confidentiality of the information. The objective of the research was also explained, and the importance of the sincerity of the answers was emphasized.

We classified students into low, moderate, and high attention, clarity, and emotional repair when their TMMS-24 scores were below 25%, between 25% and 75%, and above 75%, respectively, in the group.

### 2.4. Data Analysis

Firstly, the reliability of the TMMS-24 scale and its subscales was examined by means of Cronbach’s alpha and McDonald’s Omega. Then, in order to check whether the personal, academic, and social variables influenced each of the subscales of EI (TMMS-24), a multivariate analysis of variance (MANOVA) was performed. The following subscales of the TMMS-24 were used as dependent variables: emotional attention (EA), emotional clarity (EC), and emotional restoration (ER). Analyses were performed with the SPSS 24.0 statistical program. In this research, questionnaires that were incomplete (missing data on variables) were eliminated before the database was constructed. As a result, 69 questionnaires were not included in the study (recorded loss: 7.2%). A 95% confidence interval was used for the interpretation of all statistics.

## 3. Results

The reliability of the Trait-Meta Mood Scale (TMMS-24), measured by Cronbach’s alpha, was 0.92, and Mcdonald’s omega 0.93. The reliability of the different subscales was as follows: Cronbach’s alpha for emotional attention (EA) was 0.90 and Mcdonald’s omega was 0.92, for emotional clarity (EC) was 0.89 and Mcdonald’s omega was 0.91, and for emotional repair (ER) was 0.87, and Mcdonald’s omega was 0.90.

In the personal variables, the means and standard deviations obtained by the different groups (female and male in gender; <13 years, 13–15 years, >15 years in age; low, moderate, and high physical exercise practice) in the scores of the different subscales that make up the EI (TMMS-24): emotional attention (EA), emotional clarity (EC), and emotional repair (ER), are found in Table 1.

The MANOVA results revealed that there were significant differences between female and male individuals in EI on the scores of the different subscales of the TMMS-24 (EA, EC, ER) (Wilks’ Lambda = 0.94, F(3, 960) = 20.62, *p* < 0.001, ή^2^_p_ = 0.06, power = 1); age groups (<13 years, 13–15 years, >15 years) on the scores of the different subscales of the TMMS-24 (EA, EC, ER) (Wilks’ Lambda = 0.98, F(6, 959) = 2.68, *p* < 0.05, ή^2^_p_ = 0.01, power = 0.87); and the low, medium and high physical exercise groups on the different subscales of the TMMS-24 (EA, EC, ER) (Wilks’ Lambda = 0.95, F(6, 916) = 7.58, *p* < 0.001, ή^2^_p_ = 0.02, power = 1).

Univariate analyses (Table 2) indicated that there were significant differences in gender, age, and physical exercise practice with the different subscales of the TMMS-24 (EA, EC, ER). Gender differences were significant for emotional attention (F(1, 962) = 19.11, *p* < 0.001, ή^2^_p_ = 0.02, power = 0.99), emotional clarity (F(1, 962) = 13.76, *p* < 0.001, ή^2^_p_ = 0.01, power = 0.96), and emotional repair (F(1, 962) = 18.09, *p* < 0.001, ή^2^_p_ = 0.02, power = 0.99). Tukey’s post hoc analysis revealed that female gender individuals had better emotional attention (*M* = 2.06, *SD* = 0.97) than male gender individuals (*M* = 1.87, *SD* = 0.69), while male gender individuals had better emotional clarity (*M* = 2.02, *SD* = 0.69) and emotional repair (*M* = 2.05, *SD* = 0.69) than female gender individuals (EC: *M* = 1.85, *SD* = 0.69; ER: *M* = 1.86, *SD* = 0.71).

Age differences were not significant for emotional attention (F(3, 960) = 0.98, *p* > 0.05, ή^2^_p_ = 0.002, power = 0.22) and emotional clarity (F(3, 960) = 2.40, *p* > 0.05, ή^2^_p_ = 0.005, power = 0.48), but there were significant differences in emotional repair (F(6, 959) = 5.67, *p* < 0.01, ή^2^_p_ = 0.012, power = 0.86). Tukey’s post hoc analysis revealed that younger individuals (<13 years) had better emotional repair (*M* = 2.05, *SD* = 0.70) than middle-aged (13–15 years: *M* = 1.90, *SD* = 0.71) and older individuals (>15 years: *M* = 1.87, *SD* = 0.71).

Differences in physical exercise practice were significant for emotional mindfulness (F(1, 276) = 3.46, *p* = 0.03, ή^2^_p_ = 0.07, power = 0.65), emotional clarity (F(1, 276) = 12.48, *p* < 0.001, ή^2^_p_ = 0.026, power = 0.99), and emotional repair (F(1, 276) = 16.02, *p* < 0.001, ή^2^_p_ = 0.034, power = 1). Tukey’s post hoc analysis revealed that individuals engaging in high physical exercise (*M* = 2.07, *SD* = 0.72) had better emotional mindfulness than those engaging in low physical exercise (*M* = 1.91, *SD* = 0.70), those engaging in high (*M* = 2.01, *SD* = 0.74) or moderate physical exercise (*M* = 1.99, *SD* = 0.65) had better emotional clarity than low exercisers (*M* = 1.73, *SD* = 0.71), and high (*M* = 2.11, *SD* = 0.74) or moderate exercisers (*M* = 1.99, *SD* = 0.67) had better emotional repair than low exercisers (*M* = 1.75, *SD* = 0.72).

In the academic variables, the means and standard deviations obtained by the different groups (school year: 1st CSE, 2nd CSE, 3rd CSE, 4th CSE; academic record: low, medium, high; extracurricular activities attendance: yes, no) in the scores of the different subscales that make up EI (TMMS-24): emotional attention (AE), emotional clarity (EC), and emotional repair (ER) can be found in Table 3.

The results of the MANOVA indicated that there were no significant differences between individuals in the first, second, third, or fourth grade of CSE in the scores of the subscales of the TMMS-24: emotional attention (EA), emotional clarity (EC), and emotional repair (ER), Wilks’ Lambda = 0.98, F(3, 957) = 1.63, *p* = 0.08, ή^2^_p_ = 0.01, power = 0.80. There were also no significant differences between individuals who did or did not engage in after-school activities on the TMMS-24 subscale scores: emotional attention (EA), emotional clarity (EC), and emotional repair (ER), Wilks’ Lambda = 0.99, F(3, 960) = 2.19, *p* = 0.09, ή^2^_p_ = 0.01, power = 0.56. However, significant differences existed between individuals with low, medium, or high academic records on the TMMS-24 subscale scores: emotional attention (EA), emotional clarity (EC), and emotional repair (ER), Wilks’ Lambda = 0.98, F(6, 959) = 2.19, *p* < 0.05, ή^2^_p_ = 0.01, power = 0.78.

Univariate analyses (Table 4) revealed that academic record was not significant for emotional attentiveness (F(2, 961) = 0.18, *p* = 0.08, ή^2^_p_ = 0.01, power = 0.08) nor for emotional clarity (F(2, 961) = 0.57, *p* = 0.56, ή^2^_p_ = 0.03, power = 0.14). However, academic record was significant for emotional repair (F(2, 961) = 4.63, *p* < 0.05, ή^2^_p_ = 0.012, power = 0.78). Tukey’s post hoc analysis revealed that individuals with a good academic record (*M* = 2.06, *SD* = 0.71) had significantly greater emotional repair than those with a low academic record (*M* = 1.87, *SD* = 0.70).

Finally, in the social variables, the means and standard deviations obtained by the different groups (low, moderate, and high social ability) in the scores of the different subscales that make up EI (TMMS-24): emotional attention (EA), emotional clarity (EC), and emotional repair (ER), can be found in Table 5.

MANOVA results revealed significant differences between social ability categories (low, moderate, high) and dependent variables (TMMS-24 subscales: EA, EC, ER) (Wilks’ Lambda = 0.86, F(5, 272) = 24.97, *p* < 0.001, ή^2^_p_ = 0.07, power = 1). Univariate analyses (Table 6) showed that differences in social skills were significant for emotional attention (F(1, 276) = 8.52, *p* < 0.001, ή^2^_p_ = 0.017, power = 0.967), emotional clarity (F(1, 276) = 37.89, *p* < 0.001, ή^2^_p_ = 0.073, power = 1), and emotional repair (F(1, 276) = 66.46, *p* < 0.001, ή^2^_p_ = 0.122, power = 1). Tukey’s post hoc analyses revealed that: individuals with high (M = 2.07, SD = 0.69) and moderate (*M* = 1.99, *SD* = 0.66) social skill had better emotional attention than those with low social skill (*M* = 1.82, *SD* = 0.72); individuals with high (*M* = 2.17, *SD* = 0.72) and moderate (*M* = 1.96, *SD* = 0.66) social skill had better emotional clarity than those with low social skill (*M* = 1.64, *SD* = 0.64), and those with high social skill (*M* = 2. 17, *SD* = 0.72) had better emotional clarity than those of moderate social ability (*M* = 1.96, *SD* = 0.66); and individuals with high (*M* = 2.22, *SD* = 0.72) and moderate (*M* = 2.02, *SD* = 0.67) social skill had better emotional repair than those with low social ability (*M* = 1.55, *SD* = 0.59), and those with high social ability (*M* = 2.22, *SD* = 0.72) had better emotional repair than those with moderate social ability (*M* = 2.02, *SD* = 0.67).

## 4. Discussion and Conclusions

EI has aroused great interest in the socio-educational field, as it is a key component for the physical, psychological, and social well-being of the individual. Consequently, the main objective of the study was to determine whether personal, academic, and social variables influenced EI in adolescent populations. Thus, in general, the results obtained have evidenced the relevant contribution of personal, academic, and social variables on attention, clarity, and emotional repair in adolescence. These data are in line with previous studies [48,49,50,51,52,53].

Regarding the instrument used (TMMS-24), previous evidence demonstrates good reliability and validity of the instrument [54,55,56,57,58], offering in this work excellent reliability (Cronbach’s alpha = 0.92; Mcdonald’s omega 0.93) in line with previous studies carried out.

Firstly, taking into account the personal variables, the results confirm our first hypothesis (H1), as these variables have a significant influence on emotional intelligence. In the personal variables, it was found that gender significantly influenced attention, clarity, and emotional repair. Girls had better emotional attention, while boys achieved better emotional clarity and repair. Numerous investigations have considered that the relationship between gender and EI is still unclear. These data are consistent with the research conducted by Martínez et al. [59] and Rodríguez et al. [60] in pointing out that women reach higher levels of attention towards emotions (especially regarding their personal relationships). Along the same lines, research conducted by Cerón et al. [61], Gartzia et al. [62], Estrada [63], and Ruiz and Carranza [16] confirms that women tend to outperform men in different dimensions of EI, but do not do so in the complete EI scores. That is, it seems that the female gender has a greater awareness of their emotions, of recognizing their own feelings, and of knowing what they mean. On the contrary, the male gender has a greater ability to know and understand their emotions, understand their evolution, and integrate them into their thoughts, as well as have a better ability to regulate and control positive and negative emotions.

Regarding age, the only significant influence was found in emotional repair, but no significant differences were found in attention and emotional clarity: the younger the age, the better the emotional repair. Similarly, no significant differences were found between the realization or not of extracurricular activities in the three scales of the TMMS-24 (attention, clarity, and emotional regulation). In this same dynamic, Jiménez et al. [64] indicate that no significant differences are observed in terms of age in EI. However, other studies carried out highlight that age is a determining factor, with a progressive growth that causes important changes in all the capacities that are part of EI [65,66].

Secondly, considering the school variables, the data do not validate our second hypothesis (H2) since only the school year was statistically significant. In relation to physical exercise, the results revealed differences between the different groups on the EI subscales. Students with a high level of physical exercise were found to have better scores on emotional attention, emotional clarity, and emotional repair than those with less exercise. In this sense, the literature has shown positive relationships between EI and physical activity variables. Thus, positive relationships have been established between levels of physical activity practice and EI [18,67,68]. Similarly, participation in yoga [69], biodance [70], or body expression programs [71] have been shown to be effective for the development of EI.

In relation to academic variables, no significant differences have been found between the different ESO courses and the subscales of EI (attention, clarity, and repair). However, significant differences were found between a poor, fair, or good academic record and emotional regulation. Students with good academic records had greater emotional repair than those with low academic records.

Fernández-Lasarte, Ramos-Díaz, and Axpe [72] highlight the explanatory capacity of emotional repair on academic performance. Likewise, Guil and Gil-Olarte [73] argue that high EI is related to and predicts better academic performance. In fact, there are numerous studies that have found a positive association with the high predictive capacity of EI on the academic performance of students in all their educational stages [27,74,75,76,77]. However, there are also some studies, such as those conducted by Mavroveli et al. [78] or Antonio-Agirre et al. [27], that have provided evidence that makes clear the absence of a direct relationship between perceived EI and academic performance.

Finally, the results confirm our third hypothesis for social skills (H3). Consequently, significant differences were found between low, moderate, or high social ability in the three subscales that make up EI (attention, clarity, and restoration). Thus, adolescents with high or moderate social ability had better attention, clarity, and emotional repair. In other words, better social skills also increase EI in adolescent populations.

There are numerous studies that corroborate that an increase in the development of social skills promotes an increase in EI [79,80,81]. Thus, empirical research findings support that EI is a key variable in the maintenance of positive social interactions [25,38,82]. Consequently, it can be affirmed that there is a significant relationship between EI and social skills [83,84].

Among the limitations of the study, it should be noted that only the self-report questionnaire was used as a measurement instrument, which may generate biases due to fatigue of some individuals or problems such as social desirability and sincerity. In conclusion, although the variables analyzed do not reflect all the changes that affect the development of EI, it is true that an evident influence of these variables on attention, clarity, and emotional repair in the adolescent period can be observed.

## Figures and Tables

**Table 1:** Means and standard deviations of the subscales of the TMMS-24 questionnaire as a function of gender, age, and physical exercise.

**Gender**	**Female**	**Male**		**Total**
EA	2.06 (0.67)	1.86 (0.70)		1.96 (0.69)
EC	1.85 (0.69)	2.02 (0.70)		1.93 (0.70)
ER	1.85 (0.72)	2.05 (0.69)		1.95 (0.71)
**Age**	**<13 years**	**13–15 years**	**>15 years**	**Total**
EA	1.96 (0.67)	1.95 (0.72)	2.03 (0.66)	1.97 (0.69)
EC	2.01 (0.71)	1.89 (0.69)	1.91 (0.68)	1.93 (0.70)
ER	2.05 (0.70)	1.90 (0.72)	1.88 (0.71)	1.94 (0.71)
**Physical Exercise**	**Low**	**Moderate**	**High**	**Total**
EA	1.91 (0.70)	1.96 (0.67)	2.07 (0.73)	1.97 (0.69)
EC	1.73 (0.71)	1.99 (0.66)	2.01 (0.74)	1.93 (0.70)
ER	1.75 (0.72)	1.98 (0.67)	2.11 (0.74)	1.96 (0.71)

EA—emotional attention; EC—emotional clarity; ER—emotional repair.

**Table 2:** Multivariate analysis of variance (MANOVA). Independent variables: gender, age, and physical exercise practice. Dependent variables: subscales of EI (TMMS-24).

Independent Variables	Subscales EI	F	*p*	η_p_^2^	Power
Gender	EA	19.11	0.000	0.02	0.99
	EC	13.76	0.000	0.01	0.96
	ER	18.09	0.000	0.02	0.99
Age	EA	0.98	0.375	0.01	0.22
	EC	2.39	0.091	0.01	0.49
	ER	5.66	0.004	0.01	0.86
Physical exercise	EA	3.46	0.032	0.01	0.65
	EC	12.48	0.000	0.03	0.99
	ER	16.02	0.000	0.03	1

EI—emotional intelligence; EA—emotional attention; EC—emotional clarity; ER—emotional repair.

**Table 3:** Means and standard deviations of the subscales of the TMMS-24 questionnaire according to school year, academic record, and attendance to extracurricular activities.

**School Year**	**1st CSE**	**2nd CSE**		**3rd CSE**	**4th CSE**	**Total**
EA	2.01 (0.70)	1.81 (0.65)		1.86 (0.68)	1.91 (0.63)	1.89 (0.67)
EC	1.90 (0.75)	1.93 (0.69)		1.78 (0.67)	1.72 (0.66)	1.83 (0.69)
ER	2.13 (0.66)	1.95 (0.71)		1.93 (0.72)	1.91 (0.70)	1.98 (0.71)
**Academic Record**	**Low**		**Medium**		**High**	**Total**
EA	1.96 (0.68)		1.98 (0.69)		1.94 (0.70)	1.97 (0.69)
EC	1.94 (0.72)		1.91 (0.67)		1.96 (0.71)	1.93 (0.69)
ER	1.87 (0.70)		1.94 (0.71)		2.07 (0.71)	1.95 (0.71)
**Extracurricular Activities**	**Yes**			**No**		**Total**
EA	1.95 (0.69)			2.04 (0.68)		1.97 (0.69)
EC	1.93 (0.70)			1.92 (0.70)		1.93 (0.69)
ER	1.97 (0.71)			1.87 (0.72)		1.95 (0.71)

EA—emotional attention; EC—emotional clarity; ER—emotional repair; CSE—Compulsory Secondary Education.

**Table 4:** Multivariate analysis of variance (MANOVA) of school year, academic record, and extracurricular activities on EI (TMMS-24: subscales attention, clarity, and emotional repair).

Independent Variable	Subscales EI	F	*p*	η_p_^2^	Power
Academic record	EA	0.18	0.837	0.01	0.08
	EC	0.57	0.564	0.03	0.14
	ER	4.63	0.01	0.12	0.78

EI—emotional intelligence; EA—emotional attention; EC—emotional clarity; ER—emotional repair.

**Table 5:** Means and standard deviations of the subscales of the TMMS-24 questionnaire as a function of social skills.

Social Skills	Low	Moderate	High	Total
EA	1.82 (0.72)	1.99 (0.66)	2.07 (0.69)	1.97 (0.69)
EC	1.64 (0.64)	1.96 (0.66)	2.17 (0.72)	1.93 (0.69)
ER	1.55 (0.59)	2.02 (0.68)	2.22 (0.72)	1.95 (0.71)

EA—emotional attention; EC—emotional clarity; ER—emotional repair.

**Table 6:** Multivariate analysis of variance (MANOVA) of social skills in EI (TMMS-24: subscales attention, clarity, and emotional repair).

Independent Variable	Subscales EI	F	*p*	η_p_^2^	Power
Social skills	EA	8.52	0.000	0.02	0.98
	EC	37.89	0.000	0.07	1
	ER	66.46	0.000	0.12	1

EI—emotional intelligence; EA—emotional attention; EC—emotional clarity; ER—emotional repair.

## Data Availability

The data are not publicly available due to confidentiality reasons.
